# Risk factors for adolescent smoking uptake: Analysis of prospective data from the UK Millennium Cohort Study

**DOI:** 10.18332/tid/152321

**Published:** 2022-10-01

**Authors:** Charlotte Vrinten, Jennie C. Parnham, Filippos T. Filippidis, Nicholas S. Hopkinson, Anthony A. Laverty

**Affiliations:** 1 Public Health Policy Evaluation Unit, School of Public Health, Imperial College London, London, United Kingdom; 2 National Heart and Lung Institute, Royal Brompton Hospital, Imperial College London, London, United Kingdom

**Keywords:** adolescent, cohort studies, peer group, social media, smoking, tobacco use

## Abstract

**INTRODUCTION:**

Most people who smoke initiate smoking in adolescence. Risk factors for smoking are changing over time as demographics shift, and technologies such as social media create new avenues for the tobacco industry to recruit smokers. We assessed risk factors associated with smoking uptake and regular smoking among a representative cohort of UK adolescents.

**METHODS:**

Data come from 8944 children followed prospectively as part of the UK Millennium Cohort Study. Smoking uptake was assessed as adolescents who had never smoked tobacco at the age of 14 years, but reported smoking ≥1 cigarette per week by the age of 17 years (regular smoking). We used logistic regression to assess associations between smoking uptake and selected sociodemographic factors including household income, caregiver smoking, peer smoking, and social media use. Weighted percentages and Office for National Statistics Data were used to estimate numbers of regular smokers and new smokers in the UK.

**RESULTS:**

Among the whole sample, 10.6% of adolescents were regular smokers at the age of 17 years. Of these, 52% initiated smoking between the ages of 14 and 17 years. Uptake was more common if caregivers smoked (13.6% vs 5.0%, p<0.001) or friends smoked (12.6% vs 4.3%, p<0.001), and among those reporting >5 hours/day of social media use (9.8% vs 4.1%, p=0.006). Applying these percentages to population data, an estimated 160000 adolescents in the UK were regular smokers by the age of 17 years, of whom more than 100000 initiated smoking between the ages of 14 and 17 years.

**CONCLUSIONS:**

This analysis of smoking uptake and regular smoking highlight that smoking behavior remains highly transmissible within families and peer groups, reinforcing inequalities. Social media are highlighted as a potential vector.

## INTRODUCTION

Most people who smoke initiate smoking during their teenage years, and earlier uptake of smoking is linked to being more likely to smoke in later years^1^. Additionally, inequalities between groups in smoking uptake are an important driver of inequalities in tobacco-related health outcomes^2^. The UK Government is committed to achieving a ‘smoke-free generation’, and preventing uptake among adolescents will be a key factor to achieve this^3^. The Children’s Charter for Lung Health includes addressing child smoking as one of its key elements^4^. Previous analysis of the UK Millennium Cohort Study (MCS), using data collected between 2011 and 2014, identified that caregiver and peer smoking were important factors associated with smoking uptake by the age of 14 years^5^. As data are now available on this cohort up to the age of 17 years, it is possible to investigate factors associated with continued or new uptake of smoking in late adolescence.

In the present study, we extend previous analyses by: 1) assessing levels and risk factors for smoking in late adolescence (17 years), 2) investigating risk factors for smoking uptake between early and late adolescence; and 3) presenting regional estimates of smoking and smoking uptake among late adolescents.

## METHODS

The MCS is a birth cohort study which follows children born between September 2000 and January 2002^6^. We used data collected from both children and their main caregiver within the household at waves six and seven. At wave six, the majority of children (73.5%) were 14 years, although this ranged from 13 to 15 years depending on birth date and date of fieldwork. For ease of reference, we refer to children in this wave as ‘aged 14 years’. Similarly, the majority of children (66.9%) were aged 17 years at wave seven (range: 16–18), and we refer to them as ‘aged 17 years’; a total of 9848 children participated in both waves. After excluding those with missing data, 8944 (90.8%) individuals were available for analyses.

Smoking status at waves six and seven was assessed by asking children to select one of six statements that best described them: ‘I have never smoked cigarettes’, ‘I have only ever tried smoking cigarettes once’, ‘I used to smoke sometimes but I never smoke a cigarette now’, ‘I sometimes smoke cigarettes now but I don’t smoke as many as one a week’, ‘I usually smoke between one and six cigarettes a week’, and ‘I smoke more than six cigarettes a week’. Regular smoking at the age of 17 years was defined as those who reported smoking at least one cigarette per week at wave seven. Smoking uptake between the ages of 14 and 17 years was defined as those who reported ‘never’ smoking at the age of 14 years (wave six) and regular smoking at the age of 17 years (wave seven).

In separate logistic regression models, we assessed associations of age, gender, ethnicity, household income, country, caregiver current smoking, peer smoking, and social media use with regular smoking at the age of 17 years and with smoking uptake of people aged between 14 and 17 years.

We estimated national numbers of smoking at the age of 17 years and smoking uptake between 14 and 17 years using data on population size by age from the Office for National Statistics (ONS). We used survey weights generated by the survey team to adjust for non-response bias and sampling.

Further details on survey questions and producing national estimates are given in the Supplementary file. We also present additional analyses of the associations of social media use at the age of 17 years and transitions in social media use between 14 and 17 years with smoking (Supplementary file). Finally, we present analyses of the interactions of household income with caregiver smoking, peer smoking, and social media use (Supplementary file).

## RESULTS

About one in ten participants (10.6%) reported regular smoking at the age of 17 years (Table 1 and Supplementary file Table 1). Of these, 52% initiated smoking between the ages of 14 and 17 years, 11% were already smoking regularly at the age of 14 years, and 37% had tried smoking or smoked less than one cigarette per week at 14 years. Of the never smokers at the age of 14 years, 6.3% (n=488) reported regular smoking (i.e. at least one cigarette per week) at 17 years (Table 1 and Supplementary file Table 1).

**Table 1:** Adjusted logistic regression analyses of regular smoking at the age of 17 years and smoking uptake between the ages of 14 and 17 years

	*Regular smoking at the age of 17 years (n=8944)*		*Smoking uptake between the ages of 14 and 17 years (n=7786)* ^*b*^	
	*%*	*AOR (95% CI)* ^*a*^	*%*	*AOR (95% CI)* ^*a*^
**All**	10.6	-	6.3	-
**Age** (years)				
16 (Ref.)	9.3	1	5.5	1
17–18	11.3	0.97 (0.78–1.21)	6.6	0.94 (0.71–1.25)
**Gender**				
Male (Ref.)	10.6	1	6.7	1
Female	10.6	0.90 (0.70–1.16)	5.9	0.81 (0.58–1.12)
**Ethnicity**				
White (Ref.)	12.0	1	7.3	1
Mixed	10.5	1.04 (0.57–1.92)	3.7	0.64 (0.26–1.58)
Indian	3.1	**0.35 (0.15–0.82)**	2.1	0.44 (0.16–1.25)
Pakistani/Bangladeshi	3.1	**0.17 (0.10–0.31)**	1.9	**0.17 (0.07–0.38)**
Black or Black British	2.8	**0.27 (0.12–0.64)**	1.1	**0.22 (0.06–0.74)**
Other	6.3	0.78 (0.38–1.59)	3.7	0.75 (0.30–1.91)
**Household income**				
Q1 (highest) (Ref.)	7.2	1	4.3	1
Q2	8.8	**1.40 (1.01–1.94)**	5.3	**1.66 (1.07–2.57)**
Q3	11.3	1.32 (0.97–1.79)	7.8	**1.64 (1.19–2.26)**
Q4	15.9	**2.30 (1.73–3.06)**	8.7	**2.39 (1.64–3.47)**
Q5 (lowest)	13.0	**1.79 (1.25–2.56)**	7.0	**1.96 (1.16–3.29)**
**Country**				
England (Ref.)	10.2	1	5.7	1
Wales	11.4	0.84 (0.57–1.23)	7.5	1.03 (0.64–1.66)
Scotland	12.8	1.22 (0.95–1.58)	7.7	1.19 (0.84–1.69)
Northern Ireland	10.1	0.97 (0.68–1.38)	7.2	0.99 (0.68–1.44)
**Caregiver smoking**				
No (Ref.)	7.9	1	5.0	1
Yes	23.2	**2.06 (1.68–2.52)**	13.6	**2.06 (1.57–2.71)**
No answer	7.5	1.28 (0.40–4.13)	3.3	0.76 (0.13–4.35)
**Peer smoking**				
None (Ref.)	5.1	1	4.3	1
At least some	22.9	**3.67 (2.78–4.85)**	12.6	**2.32 (1.62–3.32)**
No answer	7.5	1.26 (0.81–1.94)	5.4	1.01 (0.61–1.67)
**Social media use on weekdays at the age of 14 years** (hours)				
<1 (Ref.)	6.0	1	4.1	1
≥1 and <5	10.9	**1.38 (1.05–1.81)**	6.9	**1.41 (1.01–1.96)**
≥5	18.8	**1.91 (1.41–2.59)**	9.8	**1.69 (1.16–2.46)**

*Unweighted %.

a AOR: weighted adjusted odds ratio, mutually adjusted for all covariates included in the table. Significant associations in bold.

b This is a subsample of the entire sample and consists of ‘never smokers’ at the age of 14 years (i.e. it excludes those who reported any smoking at the age of 14 years).

Factors associated with being a regular smoker at the age of 17 years and taking up smoking between the ages of 14 and 17 years were similar (Table 1). Those from ethnic minority backgrounds were less likely to be regular smokers at 17 years or to take up smoking, while those from lower income households, and those with caregivers and peers who smoked were more likely to do so.

Adolescents whose caregiver was smoking when they were 14 years were more than twice as likely to be a smoker at 17 years, and to start smoking between the ages of 14 and 17 years, than those whose caregivers were not smoking. Similarly, adolescents who reported peer group smoking were more than three times as likely to smoke at 17 years and more than twice as likely to take up smoking between ages 14 and 17 years than those whose peers did not smoke (Table 1 and Supplementary file Figure 1). Both regular smoking and smoking uptake were more common among adolescents in lower income households. For example, those in the lowest household income group were almost twice as likely to take up smoking as those in the highest household income group (AOR=1.96; 95% CI: 1.16–3.29).

Those who spend 1–5 hours per day on social media were 1.4 times more likely, and those who spend more than 5 hours were almost twice as likely, to smoke at the age of 17 years or take up smoking aged between 14 and 17 years than those who spent less than 1 hour per day on social media (test for trend p<0.001 for both outcomes) (Table 1). Additional analyses on social media use (Supplementary file Table 2), showed that higher social media use at 17 years was also associated with a higher likelihood of regular smoking at 17 years and initiating smoking between the ages of 14 and 17 years. Those who reported social media use of more than 1 hour per day at both the ages of 14 and 17 years were three times more likely to smoke at the age of 17 years than those who used social media for less than one hour per day. Analyses of the interaction of household income and caregiver smoking suggest that associations between caregiver smoking may be larger among those with lower household incomes (Supplementary file Table 3). These analyses are limited, however, by small numbers and overlapping confidence intervals. Similar patterns are observed for peer smoking (Supplementary file Table 4). Analyses of the interaction between social media use and household income also suggest a more prominent association between social media use and smoking among those from lower incomes (Supplementary file Table 5).

Our weighted estimates suggest that approximately 160000 adolescents (95% CI: 146815–181811) in the UK were regular smokers by the age of 17 years, of whom more than 100000 initiated smoking between the ages of 14 and 17 years (Supplementary file Table 6). Between the countries of the UK, smoking uptake ranged from 7.0% in England to 8.6% in Wales.

## DISCUSSION

Data from the Millennium Cohort Study show that of the nearly one in ten adolescents in the UK who were regular smokers by the age of 17 years, around half (52%) initiated smoking since the age of 14 years. Caregiver smoking, peer smoking, and social media use were linked to uptake of tobacco smoking among UK adolescents.

Previous analyses of the same cohort for the age of 14 years found that 1.9%, or an estimated 39000 early teens around the UK were smokers^5^. Together, these findings indicate that a large group of UK adolescents still take up smoking despite the government’s pledge to create a ‘smoke-free generation’ and that approaches to address this need to be delivered across childhood. They also serve as a reminder of the transmissibility of the smoking epidemic with peer and caregiver smoking increasing tobacco use among adolescents. We also found that adolescents in lower income households were more likely to take up smoking and to be regular smokers. These findings highlight the inequalities in smoking harms and that an intergenerational, comprehensive approach including preventing uptake, promoting quitting, and treating dependence is needed to tackle tobacco use^7,8^.

We found a significant independent association between social media use and smoking uptake. This finding is in line with other research, mainly conducted in the USA, which has found, for example, increased susceptibility to smoking uptake and higher levels of smokeless tobacco use among children exposed to online tobacco advertising^9,10^. Although causation cannot be inferred from this, the findings do reinforce concerns that social media content may promote smoking. This study adds to an increasing evidence base, including a recent systematic review of 29 studies which identified high levels of tobacco marketing on social media, and a link between this and youth smoking^11^. It should be noted that while our study is the first reporting of data on this from the UK, we were unable to assess actual exposure to tobacco promotion on social media. This means that there may be other explanations including a clustering of unhealthy behaviors within some groups. Nonetheless, together the evidence reinforces calls for action, including those from the Royal College of Physicians to ban all social media marketing of tobacco products^12,13^. These findings also strengthen arguments that legislation to address online safety should consider public health harms, including those from tobacco advertising, and of the need for continued awareness over the changing landscape of tobacco advertising over time.

### Strengths and limitations

A strength of this study is that our covariates, such as household income, caregiver smoking, peer smoking, and social media use, were assessed prospectively, before uptake of smoking, adding strength to the temporality of the relationship between these factors and subsequent smoking uptake. Limitations to this work include that smoking measures were based on self-report, but previous studies have shown that this is a reliable indicator for the prevalence of actual smoking behaviour^14^. Furthermore, we did not consider e-cigarette use in the analyses, although an estimated 5-8% of adolescents use these^7^. Hence, we may have underestimated total use of nicotine-containing products by adolescents. Our findings regarding social media use are limited by the fact that the measure used was hours of use, and not a more specific measure such as actual exposure to pro-tobacco advertisements or messages. Finally, while cohort studies are prone to attrition over time, we used the survey weights provided to adjust for this and to ensure population-representativeness.

## CONCLUSIONS

These prospective data show that the relationship of caregiver and peer group smoking with smoking uptake persists throughout childhood, and highlights a potential role for social media as an important potential novel vector.

## Data Availability

The data supporting this research are available from the following source: beta.ukdataservice.ac.uk/datacatalogue/series/series?id=2000031.
