# Supplementary material for: Risk factors for adolescent smoking uptake: Analysis of prospective data from the UK Millennium Cohort Study
Source: Tob Induc Dis. 2022 Oct 1;20:83. doi: 10.18332/tid/152321 (PMC9521183; doi:10.18332/tid/152321)
Supplement: Supplementary file 1 [file TID-20-83-s1.pdf]

APPENDIX.

Appendix to: Charlotte Vrinten, Jennie C. Parnham, Filippos T. Filippidis, Nicholas S. Hopkinson, Anthony A. Laverty. Risk factors for adolescent smoking uptake: Analysis of prospective data from the UK Millennium Cohort Study.

Appendix contents

1. Additional description of the Millennium Cohort Study and sample selection
2. Assessment of outcomes
3. Assessment of covariates
4. Population data
5. Analyses
6. Additional analyses
7. References
8. Appendix tables

1. Additional description of the Millennium Cohort Study and sample selection

The original sample of the Millennium Cohort Study (MCS) consisted of nearly 19,000 children who were born in England, Wales, Scotland and Northern Ireland between September 2000 and January 2002. A stratified, clustered random sample design was used with oversampling from disadvantaged areas (i.e. those living in the poorest 25% of wards (or Electoral Divisions in Wales) according to the Child Poverty Index) and areas with a high proportion of ethnic minority groups (at least 30%). Once the sample wards were selected, all children on the Child Benefit register who turned 9 months olds during the survey period were invited to participate.

Seven waves of data collection have taken place since inception of the cohort, at age 9 months and 3, 5, 7, 11, 14 and 17 years. Data for wave six (14 years) were collected between Jan 2015 and March 2016. Data for wave seven (17 years) were collected between Jan 2018 and March 2019 (Fitzsimons et al., 2020a).

Wave six resulted in 11,726 families with productive interviews and 10,625 for wave seven. Almost ten thousand children completed both waves 6 and 7 (N=9,848). Of these, 901 (9.1%) had missing data on smoking at wave six and/or wave seven and three participants (0.03%) had missing data on social media use at wave six, and these were excluded from our analytical sample. This resulted in an analytical sample of N=8,944.

Descriptive statistics for the sample can be found in Appendix Table 1.

2. Assessment of outcomes

Smoking status was assessed by asking children to select one of six statements that best described them at waves six and seven: "I have never smoked cigarettes", "I have only ever tried smoking cigarettes once", "I used to smoke sometimes but I never smoke a cigarette now", "I sometimes smoke cigarettes now but I don't smoke as many as one a week", "I usually smoke between one and six cigarettes a week", and "I smoke more than six cigarettes a week".

Regular smoking at age 17 was defined as those who reported smoking at least one cigarette per week at wave seven. *Smoking uptake between age 14 and 17* was defined as those who reported "never" smoking at age 14 (wave six) and regular smoking at age 17 (wave seven).

3. Assessment of covariates

Age at wave 7, gender (wave 7), ethnicity in six groups (wave six), and UK country of residence (wave seven) were recorded in the survey. For missing data on age at wave 7 (n=107, 1.2%), gender (n=107, 1.2%), ethnicity (n=62, 0.7%), and region of residence (n=107, 1.2%), we carried forward responses from previous survey waves (for age at wave 7, this was imputed as age at wave 6 plus 3 years). For the logistic regression analyses, we collapsed the categories for age 17 and 18 at wave seven due to low numbers of participants aged 18 (n=28; 0.3%). Missing data on parental (n=62, 0.8%) and peer smoking (n=730, 8.2%) were coded as separate category for analysis.

Household income (wave six) was based on the Organisation for Economic Co-operation and Development (OECD) equivalised income and was categorised in five groups (Fitzsimons, 2020b).

Caregiver smoking (yes/no) at wave six was assessed in the interview with the cohort member's main carer.

Peer smoking at wave six was assessed in the cohort member interview with the question: "How many of your friends smoke cigarettes? Do not include e-cigarettes" with response options: "none of them", "some of them", "most of them", "all of them". These were recoded for analysis as "No" ("none of them") versus "At least some" (all other responses). Missings were recoded as a separate category for analysis.

Social media use at wave six was assessed with the question: "On a normal week day during term time, how many hours do you spend on social networking or messaging sites or apps on the internet such as Facebook, Twitter and WhatsApp?" with response options: "none", "less than half an hour", "half an hour to less than 1 hour", "1 hour to less than 2 hours", "2 hours to less than 3 hours", "3 hours to less than 5 hours", "5 hours to less than 7 hours", "7 hours or more". For wave seven, social media use was assessed with the item: "On a normal week day, how many hours do you spend on social networking or messaging sites or apps on the internet such as Facebook, Twitter, WhatsApp, Instagram and Snapchat?" with response options: "none", "less than half an hour", "half an hour to less than 1 hour", "1 hour to less than 2 hours", "2 hours to less than 3 hours", "3 hours to less than 5 hours", "5 hours to less than 7 hours", "7 hours to less than 10 hours", "10 hours or more". Responses for both waves were recoded for analysis as "less than 1 hour", "between 1 and 5 hours", and "more than 5 hours".

We examined the associations between social media use at age 14 and age 17 with being a regular smoker at age 17 and with initiating smoking between age 14 and 17. In addition, we examined the association between transitions in social media use by creating a new variable describing four possible transitions: 1) low social media use at age 14 and also at age 17, 2) low social media use at age 14 and high social media use at age 17, 3) high social media use at age 14, but low social media use at age 17, and 4) high social media use at both age 14 and 17. For the purpose of these analyses, we defined "low social media use" as less than one hour per day and "high social media use" as more than one hour per day.

4. Population data

We used mid-2018 estimates from the Office for National Statistics (ONS) on population size by single year of age and sex (Office for National Statistics, 2019). This was matched to the MCS data based on UK country of residence.

We used ONS data on population size for 16- and 17-year olds by country, and weighted percentages for regular smokers and smoking uptake among 16- and 17-year olds by country obtained from the MCS data to produce country estimates for the absolute number of adolescents (with 95% confidence intervals) who were regular smokers by age 17 and for the absolute number of adolescents (with 95% CI) who had taken up smoking between age 14 and 17.

Due to small numbers in the MCS data and consequent potential for unreliable estimates, we excluded 18 year olds from these analyses (n=28, 0.3%).

5. Analyses

We used unweighted data for the descriptive characteristics of the samples, and used the weights provided by the Millennium Cohort Study team to adjust for non-response bias (from study attrition) and sampling for the logistic regression analyses of smoking at age 17, smoking uptake between age 14 and 17, and to calculate the country estimates of smokers at age 17 and smoking uptake between age 14 and 17. The Millennium Cohort Study used a stratified sampling approach, using electoral wards as sampling clusters. Sampling weights were calculated for wave 1 based on the inverse of the sampling fraction applied to each stratum and scaled to sum to the number of wards selected in each UK country (Plewis, 2007). The non-response weights considered a number of predictors of non-response, including cohort member, maternal, main caregiver and household characteristics. Cohort member characteristics included gender, ethnic group, cognitive ability at age 5 and mental health at age 11. Maternal characteristics included age at first live birth and mental health when cohort member was aged 9 months. Main caregiver characteristics included highest educational qualification, socioeconomic status, and job status. Household characteristics included tenure, accommodation type and number of parents living in the household. Final weights were constructed by multiplying the non-response weights for wave 7 by the sampling weights from wave 1. Further details on the construction of these weights can be found in the Millennium Cohort Study Seventh Sweep (MCS7): Technical Report (Centre for Longitudinal Studies, 2019), the Millennium Cohort Study Age 17 Sweep (MCS7): User Guide (Fitzsimons et al., 2020a), and The Millennium Cohort Study: Technical Report on Sampling (Plewis, 2007).

6. Additional analyses

Appendix Table 2 shows the descriptive characteristics (unweighted) and adjusted logistic regression analyses (weighted) for social media use at age 14, social media use at age 17, and transitions of social media use between age 14 and 17, and regular smoking at age 17 and smoking uptake between age 14 and 17.

Appendix Tables 3-5 show the descriptive characteristics (unweighted) and unadjusted logistic regression analyses (weighted) for interaction effects of quintile of equivalised household income with caregiver smoking (Appendix Table 3), peer smoking (Appendix Table 4), and social media use

at age 14 (Appendix Table 5) on regular smoking at age 17 and smoking initiation between age 14 and 17.

Appendix Table 6 shows the estimates of adolescent smoking at age 17 and smoking uptake between age 14 and 17 (percentages and absolute numbers), for the four constituent countries of the UK.

Appendix Figure 1. Presents a graphical representation of the adjusted odds ratios and 95% confidence intervals of regular smoking at age 17 and smoking uptake between age 14 and 17 as reported in the main manuscript.

7. References

Centre for Longitudinal Studies, UCL Institute of Education, 2019. Millennium Cohort Study Seventh Sweep (MCS7): Technical Report. Available from: https://doc.ukdataservice.ac.uk/doc/8682/mrdoc/pdf/mcs7_technical_report.pdf Date last accessed: 3 May 2022

Fitzsimons, E., Haselden, L., Smith, K., Gilbert, E., Calderwood, L., Agalioti- Sgompou, V., Veeravalli, S., Silverwood, R., Ploubidis, G. (2020a) Millennium Cohort Study Age 17 Sweep (MCS7): User Guide. London: UCL Centre for Longitudinal Studies. Available from: https://doc.ukdataservice.ac.uk/doc/8682/mrdoc/pdf/mcs7_user_guide_age17_ed2_2020_12_08.pdf Date last accessed: 20 July 2022.

Fitzsimons, E. (2020b). Millennium Cohort Study, sixth survey 2015-2016, User Guide (2nd ed). Available from: http://doc.ukdataservice.ac.uk/doc/8156/mrdoc/pdf/mcs6_user_guide_ed2_2020-08-10.pdf Date last accessed: 20 July 2022.

Office For National Statistics (2019). Population Estimates for UK, England and Wales, Scotland and Northern Ireland: Mid-2018, using April 2019 local authority district codes. Available from: https://www.ons.gov.uk/peoplepopulationandcommunity/populationandmigration/populationestimates/datasets/populationestimatesforukenglandandwalesscotlandandnorthernireland Date last accessed: 27 April 2022

Plewis, I. (2007). Millennium Cohort Study: Technical Report on Sampling (4th ed). Available from: https://doc.ukdataservice.ac.uk/doc/4683/mrdoc/pdf/mcs_technical_report_on_sampling_4th_edition.pdf Date last accessed: 20 July 2022.

8. Appendix tables.

Appendix Table 1. Descriptive sample characteristics (unweighted) of the whole sample (N=8,944), and the subsamples of those who had never smoked at age 14 (N=7,786) and those who had ever smoked at age 14 (N=1,158).

	**All (N=8,944)**	**Never smokers age 14 (N=7,786)**	**Ever smokers age 14 (N=1,158)**
	**N (%)**	**N (%)**	**N (%)**
Age at wave 7			
16	2,933 (32.8)	2,604 (33.4)	329 (28.4)
17/18	6,011 (67.2)	5,182 (66.6)	829 (71.6)
Gender			
Male	4,331 (48.4)	3,829 (49.2)	502 (43.4)
Female	4,613 (51.6)	3,957 (50.8)	656 (56.7)
Ethnicity			
White	7,087 (79.2)	6,117 (78.6)	970 (83.8)
Mixed	420 (4.7)	352 (4.5)	68 (5.9)
Indian	257 (2.9)	243 (3.1)	14 (1.2)
Pakistani and Bangladeshi	669 (7.5)	617 (7.9)	52 (4.5)
Black or Black British	290 (3.2)	265 (3.4)	25 (2.2)
Other	221 (2.5)	192 (2.5)	29 (2.5)
Household income			
Q1 (highest)	2,263 (25.3)	2,072 (26.6)	191 (16.5)
Q2	2,178 (24.4)	1,936 (24.9)	242 (20.9)
Q3	1,801(20.1)	1,569 (20.2)	232 (20.0)
Q4	1,375 (15.4)	1,105 (14.2)	270 (23.3)
Q5 (lowest)	1,327 (14.8)	1,104 (14.2)	223 (19.3)
Country			
England	5,970 (66.8)	5,199 (66.8)	771 (66.6)
Wales	1,191 (13.3)	1,028 (13.2)	163 (14.1)
Scotland	949 (10.6)	819 (10.5)	130 (11.2)
Northern Ireland	834 (9.3)	740 (9.5)	94 (8.1)
Parental smoking			
No	7,292 (81.5)	6,538 (84.0)	754 (65.1)
Yes	1,585 (17.7)	1,188 (15.3)	397 (34.3)
No answer	67 (0.8)	60 (0.8)	7 (0.6)
Peer smoking			
None	5,554 (62.1)	5,350 (68.7)	204 (17.6)
At least some	2,660 (29.7)	1,764 (22.7)	896 (77.4)
No answer	730 (8.2)	672 (8.6)	58 (5.0)
Social media use on weekdays			
Less than 1 hour	3,150 (35.2)	2,947 (37.9)	203 (17.5)
Between 1 and <5 hours	4,183 (46.8)	3,663 (47.1)	520 (44.9)
5 hours or more	1,611 (18.0)	1,176 (15.1)	435 (37.6)
Smoking at age 14			
Never	7,786 (87.0)	7,786 (100.0)	---
Ever smoking but not weekly	1,017 (11.4)	---	1,017 (87.8)
Regular (at least weekly)	141 (1.6)	---	141 (12.2)
Regular smoking at age 17			
No	7,996 (89.4)	7,298 (93.7)	698 (60.3)
Yes	948 (10.6)	488 (6.3)	460 (39.7)

Appendix Table 2. Descriptive characteristics and adjusted logistic regression analyses of social media use at ages 14 and 17, transitioning between low/high social media use between ages 14 and 17, and regular smoking at age 17 and smoking uptake between age 14 and 17 years (unweighted %; weighted aOR)[$]

		Regular smoking at age 17			Smoking uptake between ages 14 and 17	
	N (%) 8,944 (100.0)	N (%)	OR (95% CI)	N (%) 7,786 (100.0)	N (%)	OR (95% CI)
Social media use on weekdays at age 14						
Less than 1 hour	3,150 (35.2)	189 (6.0)	Ref	2,947 (37.9)	120 (4.1)	Ref
Between 1 and <5 hours	4,183 (46.8)	457 (10.9)	**1.38 (1.05; 1.81)**	3,663 (47.1)	253 (6.9)	**1.41 (1.01; 1.96)**
5 hours or more	1,611 (18.0)	302 (18.8)	**1.91 (1.41; 2.59)**	1,176 (15.1)	115 (9.8)	**1.69 (1.16; 2.46)**
Social media use on weekdays at age 17						
Less than 1 hour	842 (9.4)	33 (3.9)	Ref	794 (10.2)	17 (2.1)	Ref
Between 1 and <5 hours	3,117 (34.9)	202 (6.5)	1.55 (0.96; 2.50)	2,829 (36.3)	109 (3.9)	1.85 (0.94; 3.66)
5 hours or more	2,288 (25.6)	252 (11.0)	**2.20 (1.30; 3.74)**	1,938 (24.9)	123 (6.4)	**3.66 (1.72; 7.78)**
Missing	2,697 (30.2)*			2,225 (28.6)*		
Social media use transition between age 14 and 17^						
Low → Low	602 (6.7)	14 (2.3)	Ref	585 (7.5)	9 (1.5)	Ref
Low → High	1,630 (18.2)	73 (4.5)	**2.08 (1.06; 4.06)**	1,533 (27.2)	44 (2.9)	2.09 (0.83; 5.25)
High → Low	240 (2.7)	19 (7.9)	**2.58 (1.10; 6.05)**	209 (2.7)	8 (3.8)	1.92 (0.60; 6.21)
High → High	3,775 (42.2)	381 (10.1)	**3.09 (1.59; 6.02)**	3,234 (41.5)	188 (5.8)	**3.80 (1.58; 9.13)**
Missing	2,697 (30.2)*			2,225 (28.6)*		

$Adjusted odds ratio; adjusted for age, gender, ethnicity, household income, country, caregiver smoking, peer smoking. Significant values **in bold.**
*Excluded from logistic regression analyses.
^ Low social media use defined as up to 1 hr/day; high social media use as 1 hr/day or more

Appendix Table 3. Descriptive characteristics (unweighted) and unadjusted logistic regression analyses (weighted) for interaction effects of household income with caregiver smoking on regular smoking at age 17 and smoking initiation between age 14 and 17.

		Regular smoking at age 17			Smoking uptake between age 14 and 17	
	N (%)	N (%)	OR (95%CI)^	N (%)	N (%)	OR (95%CI)^
Household income*Caregiver smoking	8,877 (100.0)[%]	943 (10.6)		7,726 (100.0)[%]	486 (6.3)	
Q1 (highest)*No	2,112 (23.8)	138 (6.5)	Ref	1,942 (25.1)	77 (4.0)	Ref
Q1 (highest)*Yes	143 (1.6)	22 (15.8)	**2.61 (1.50; 4.54)**	123 (1.6)	11 (8.9)	**2.60 (1.22; 5.55)**
Q2*No	1,909 (21.5)	145 (7.6)	**1.61 (1.13; 2.28)**	1,722 (22.3)	84 (4.9)	**1.95 (1.22; 3.12)**
Q2*Yes	258 (2.9)	45(17.4)	**3.39 (2.25; 5.09)**	205 (2.7)	19 (9.3)	**3.05 (1.70; 5.47)**
Q3*No	1,467 (16.5)	136 (9.3)	**1.65 (1.18; 2.29)**	1,310 (17.0)	87 (6.6)	**1.81 (1.25; 2.63)**
Q3*Yes	325 (3.7)	66 (20.3)	**3.13 (2.02; 4.86)**	251 (3.3)	36 (14.3)	**3.71 (2.16; 6.37)**
Q4*No	928 (10.5)	97 (10.5)	**2.10 (1.52; 2.91)**	785 (10.2)	42 (5.4)	**1.57 (1.03; 2.41)**
Q4*Yes	435 (4.9)	122 (28.0)	**7.03 (4.86; 10.2)**	309 (4.00)	54 (17.5)	**7.64 (4.59; 12.71)**
Q5 (lowest)*No	876 (9.9)	60 (6.8)	1.46 (0.97; 2.18)	779 (10.1)	34 (4.4)	**1.84 (1.09; 3.12)**
Q5 (lowest)*Yes	424 (4.8)	112 (26.4)	**4.96 (3.33; 7.40)**	300 (3.9)	42 (14.0)	**3.58 (1.97; 6.49)**

[%] Reduced sample size due to excluding missing values ("no answer") for caregiver smoking status.

^OR = Odds Ratios; 95% CI = 95% Confidence Interval. Significant values in bold.

Appendix Table 4. Descriptive characteristics (unweighted) and unadjusted logistic regression analyses (weighted) for interaction effects of household income with peer smoking on regular smoking at age 17 and smoking initiation between age 14 and 17.

		**Regular smoking at age 17**			**Smoking uptake between age 14 and 17**	
	N (%)	N (%)	OR (95%CI)^	N (%)	N (%)	OR (95%CI)^
Household income*Peer smoking	8,214 (100.0)[%]	893 (10.9)		7,114 (100.0)[%]	452 (6.4)	
Q1 (highest)*None	1,554 (18.9)	53 (3.4)	Ref	1,514 (21.3)	40 (2.6)	Ref
Q1 (highest)*At least some	560 (6.8)	102 (18.2)	**6.85 (4.94; 9.52)**	413 (5.8)	43 (10.4)	**4.87 (3.01; 7.75)**
Q2*None	1,416 (17.2)	62 (4.4)	**2.17 (1.22; 3.86)**	1,374 (19.3)	49 (3.6)	**2.50 (1.28; 4.86)**
Q2*At least some	614 (7.5)	120 (19.5)	**7.94 (5.15; 12.23)**	423 (6.0)	49 (11.6)	**6.37 (3.48; 11.69)**
Q3*None	1,102 (13.4)	74 (6.7)	**2.15 (1.41; 3.27)**	1,062 (14.9)	62 (5.8)	**2.51 (1.59; 3.95)**
Q3*At least some	550 (6.7)	116 (21.1)	**7.98 (5.12; 12.42)**	369 (5.2)	50 (13.6)	**6.11 (3.67; 10.15)**
Q4*None	745 (9.1)	54 (7.2)	**3.75 (2.17; 6.49)**	698 (9.8)	42 (6.0)	**4.30 (2.25; 8.19)**
Q4*At least some	497 (6.1)	153 (30.8)	**15.42 (10.56; 22.50)**	289 (4.1)	45 (15.6)	**7.93 (4.56; 13.79)**
Q5 (lowest)*None	737 (9.0)	41 (5.6)	**2.34 (1.40; 3.91)**	702 (9.9)	36 (5.1)	**2.86 (1.63; 5.00)**
Q5 (lowest)*At least some	439 (5.3)	118 (26.9)	**10.62 (6.72; 16.78)**	270 (3.8)	36 (13.3)	**6.30 (3.18; 12.50)**

[%] Reduced sample size due to excluding missing values ("no answer") for peer smoking status.
^OR = Odds Ratios; 95% CI = 95% Confidence Interval. Significant values in bold.

Appendix Table 5. Descriptive characteristics (unweighted) and unadjusted logistic regression analyses (weighted) for interaction effects of household income with social media use at age 14 on regular smoking at age 17 and smoking initiation between age 14 and 17.

		Regular smoking at age 17			Smoking uptake between age 14 and 17	
	N (%)	N (%)	OR (95%CI)^	N (%)	N (%)	OR (95%CI)^
Household income*Social media use	8,944 (100.0)	948 (10.6)		7,786 (100.0)	488 (7.3)	
Q1 (highest)*<1hr	933 (10.4)	38 (4.1)	Ref	892 (11.5)	23 (2.6)	Ref
Q1 (highest)*1 - <5hrs	1,046 (11.7)	87 (8.3)	**1.82 (1.23; 2.70)**	953 (12.2)	51 (5.4)	**1.68 (1.00; 2.83)**
Q1 (highest)*>5hrs	284 (3.2)	37 (13.0)	**3.37 (2.09; 5.44)**	227 (2.9)	15 (6.6)	**2.62 (1.37; 5.02)**
Q2*<1hr	719 (8.0)	36 (5.0)	1.55 (0.88; 2.73)	686 (8.8)	22 (3.2)	1.28 (0.71; 2.31)
Q2*1 - <5hrs	1,089 (12.2)	95 (8.7)	**3.13 (1.95; 5.02)**	976 (12.5)	57 (5.8)	**3.80 (2.02; 7.14)**
Q2*>5hrs	370 (4.1)	60 (16.2)	**3.76 (2.41; 5.87)**	274 (3.5)	24 (8.8)	**2.80 (1.43; 5.47)**
Q3*<1hr	586 (6.6)	38 (6.5)	**1.78 (1.07; 2.96)**	552 (7.1)	29 (5.3)	**2.40 (1.32; 4.34)**
Q3*1 - <5hrs	858 (9.6)	97 (11.3)	**2.51 (1.63; 3.86)**	748 (9.6)	62 (8.3)	**2.56 (1.51; 4.35)**
Q3*>5hrs	357 (4.0)	68 (19.0)	**5.36 (3.18; 9.02)**	269 (3.5)	32 (11.9)	**4.54 (2.60; 7.93)**
Q4*<1hr	430 (4.8)	43 (10.0)	**3.95 (2.24; 6.98)**	387 (5.0)	29 (7.5)	**4.87 (2.39; 9.92)**
Q4*1 - <5hrs	627 (7.0)	103 (16.4)	**4.89 (3.43; 6.98)**	513 (6.6)	48 (9.4)	**3.60 (2.22; 5.83)**
Q4*>5hrs	318 (3.6)	73 (23.0)	**8.25 (5.36; 12.69)**	205 (2.6)	19 (9.3)	**4.80 (1.97; 11.70)**
Q5 (lowest)*<1hr	482 (5.4)	34 (7.1)	**2.06 (1.19; 3.58)**	430 (5.5)	17 (4.0)	1.90 (0.88; 4.13)
Q5 (lowest)*1 - <5hrs	563 (6.3)	75 (13.3)	**4.42 (2.76; 7.06)**	473 (6.1)	35 (7.4)	**3.84 (1.97; 7.48)**
Q5 (lowest)*>5hrs	282 (3.2)	64 (22.7)	**7.13 (4.36; 11.66)**	201 (2.6)	25 (12.4)	**4.94 (2.45; 9.93)**

^OR = Odds Ratios; 95% CI = 95% Confidence Interval. Significant values in bold.

Appendix Table 6. Country estimates for regular smoking at age 17 and smoking uptake between age 14 and 17 (using weighted data).

	Adolescents who are regular smokers at age 17		Adolescents taking up smoking between age 14 and 17 *	
Country/Region	% (95% CI)^	N (95% CI)^	% (95% CI)^	N (95% CI)^
England	11.4 (10.0; 12.9)	137961 (120839; 155082)	7.0 (5.7; 8.2)	83878 (68452; 99303)
Wales	11.0 (8.4; 13.5)	7359 (5643; 9075)	8.6 (5.9; 11.3)	5760 (3924; 7595)
Scotland	13.0 (10.6; 15.4)	14213 (11572; 16854)	8.0 (6.0; 10.0)	8740 (6540; 10940)
Northern Ireland	10.8 (8.5; 13.2)	4863 (3798; 5928)	7.2 (5.1; 9.2)	3219 (2302; 4136)
Overall	11.5 (10.3; 12.7)	164313 (146815; 181811)	7.1 (6.0; 8.2)	101715 (85994; 117435)

*These estimates are for "never smokers" at age 14 who reported regular smoking at age 17.

^ 95% CI = 95% confidence interval

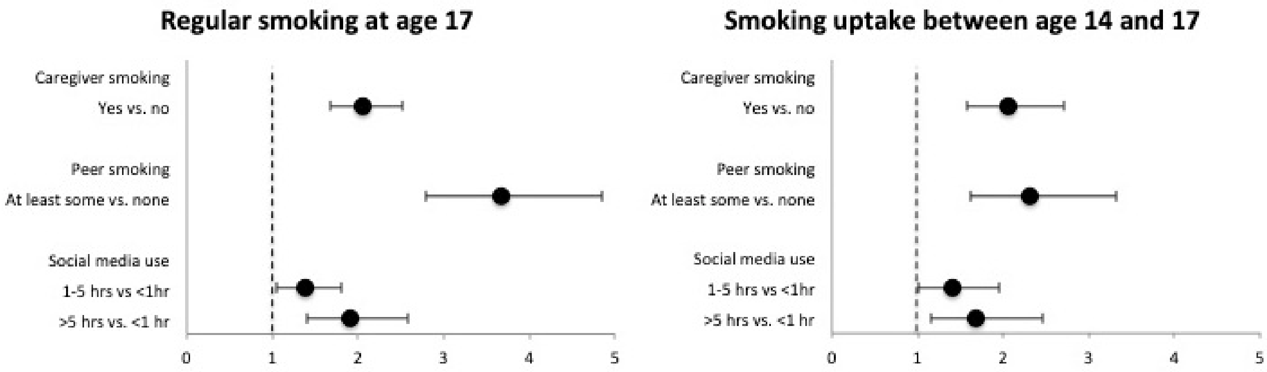

Appendix Figure 1. Adjusted odds ratios and 95% confidence intervals of regular smoking at age 17 and smoking uptake between age 14 and 17.

© 2022 Vrinten C. et al.
